# Silicon Photonic On-Chip Spectrometer Based on Cascaded Mach–Zehnder Interferometer

**DOI:** 10.3390/s26051470

**Published:** 2026-02-26

**Authors:** Yating Cui, Ye Yuan, Zan Zhang, Beiju Huang

**Affiliations:** 1 State Key Laboratory of Optoelectronic Materials and Devices, Institute of Semiconductors, Chinese Academy of Sciences, Beijing 100083, China; cuiyating@semi.ac.cn; 2 School of Electronic, Electrical and Communication Engineering, University of Chinese Academy of Sciences, Beijing 100049, China; 3 Faculty of Electronic and Information Engineering, Xi’an Jiaotong University, Xi’an 710049, China; yeyuan@stu.xjtu.edu.cn; 4 School of Electronic and Control Engineering, Chang’an University, Xi’an 710064, China; 5 College of Materials Science and Opto-Electronic Technology, University of Chinese Academy of Sciences, Beijing 100049, China

**Keywords:** computational spectrometer, Mach–Zehnder interferometer, spectral reconstruction

## Abstract

Spectrometers are essential tools for revealing the interaction between light and matter and analyzing the composition and state of materials, widely employed in scientific research, industrial inspection, and biomedicine applications. With the continuous expansion of application scenarios, higher demands are placed on the miniaturization, integration, and portability of spectrometers. This paper proposes and implements a reconfigurable silicon photonic on-chip spectrometer based on cascaded multi-stage Mach–Zehnder interferometers (MZIs). This structure achieves efficient sampling of the input spectrum by applying adjustable phase shifts to each MZI stage to construct different spectral responses. Combined with a convex optimization algorithm incorporating differential operators, the unknown input signals are decomposed into sparse and smooth components, achieving high-accuracy reconstruction. Experimental results show that the proposed five-stage MZI design with a total of 216 sampling channels achieves a spectral reconstruction resolution of 5 pm over the wavelength range from 1500 nm to 1600 nm. Moreover, the spectrometer exhibits consistently low reconstruction errors for broadband spectra, sparse spectra, and their hybrid spectral profiles. This research demonstrates excellent comprehensive performances in device structure design, phase modulation strategy, and reconstruction algorithm, providing an effective solution for realizing low-power, small-footprint, and high-precision on-chip spectral analysis.

## 1. Introduction

Spectroscopic analysis, as an fundamental tool for investigating light–matter interactions, plays an irreplaceable role in a wide range of applications, including material characterization [1], biomedical research [2], the food industry [3] and remote sensing [4]. Conventional spectrometers typically rely on dispersive or interferometric components such as gratings [5], prisms [6,7], and interferometers [8], combined with precise mechanical structures to achieve high spectral resolution and measurement stability. However, these systems are often bulky, mechanically complex, and costly, making it difficult for them to meet the growing demands of emerging application scenarios such as real-time sensing and portable devices [9,10], which severely limits their application in daily life. Computational spectrometers provide an alternative paradigm for spectral measurement [11,12,13,14,15]. The core principle lies in encoding the incident spectrum through intrinsic or tunable optical responses of the device, and then recovering the high-resolution spectrum from the limited measurements using reconstruction algorithms. Compared with traditional dispersive spectrometers, computational spectrometers offer significant advantages in terms of a reduced footprint and enhanced portability. With the rapid advancement of silicon photonics, computational on-chip spectrometers based on silicon platforms [16] have attracted increasing research interest, owing to their compact size, high level of integration, and low fabrication cost.

Existing silicon photonic computational spectrometers can generally be categorized into three main architectures: the multimode waveguide type, disordered scattering medium type, and waveguide filter type. Multimode waveguide spectrometers [17,18,19,20] utilize the difference in propagation constants among supported modes in multimode interference to sample the incident spectrum. While this approach can achieve a relatively high spectral resolution, sufficiently complex interference modes typically require long propagation lengths, resulting in a large device footprint. In addition, the limited number of output channels—the spectrometers in References [18,19] employ only 12 and 4 channels, respectively—leads to a limited operational optical bandwidth. To address this issue, Tsang [20] et al. introduced multiple MZI optical switches, increasing the number of sampling states from six physical channels to 384 programmable configurations, thereby enabling spectral reconstruction over a bandwidth of 100 nm. Compared with multimode waveguides, spectrometers based on disordered scattering media [21,22,23] have a smaller footprint, but the multiple scattering process inevitably introduces significant optical loss. For instance, the spectrometer designed by Hartmann et al. [23], which combines an efficient broadband fiber-to-chip coupling approach with a scattering region, has a transmission loss of up to a mean value of 39 dB for the visible range. Similarly, Cao et al. [21] demonstrated a spectrometer based on multiple scattering in a disordered structure. Even with the incorporation of photonic-crystal boundaries to enhance optical confinement and collection efficiency, approximately 40% of the optical power was still not captured by the photodetectors, which ultimately limited the achievable resolution of spectral reconstruction. Waveguide filter spectrometers [11,12,13,14,24,25,26,27,28] offer superior design flexibility and have attracted significant research attention in recent years. Utilizing mature silicon photonic waveguide devices such as microring resonators (MRRs), Mach–Zehnder interferometers (MZIs), and distributed Bragg reflectors (DBRs), researchers can design diverse integrated silicon photonic architectures, including reconfigurable networks, programmable photonic circuits, and waveguide filter arrays. These flexible designs, after optimization, can achieve higher sampling efficiencies [14,24]. Furthermore, with the assistance of thermo-optic phase shifters, waveguide filters can dynamically reconfigure their filtering response, resulting in an exponential increase in the number of effective sampling channels [12,25,26]. As a result, broadband and high-resolution spectral reconstruction can be realized within a limited device footprint. The key characteristics and performance comparisons of various types of spectrometers are shown in Table 1.

Based on the aforementioned background, we propose and experimentally demonstrate a reconfigurable on-chip spectrometer implemented on a silicon photonic platform. The proposed scheme is centered on a five-stage cascaded Mach–Zehnder interferometer (MZI) structure with distinct arm-length differences, forming a programmable silicon photonic filter that functions as a dynamic spectral sampling unit. By integrating tunable phase shifters into each stage, the transmission response of the filter can be flexibly reconfigured, enabling diverse spectral sampling of the incident signal. Moreover, the device incorporates two output ports, which improves the sampling efficiency and contributes to enhanced reconstruction performance. Compared with previously reported programmable photonic filters employed for spectrometer applications, the proposed architecture significantly reduces design complexity, as it avoids the need for joint optimization of numerous device parameters and coupling conditions, thereby providing a more scalable and practical solution. To balance the number of sampling channels, reconstruction accuracy, and tuning complexity, a hybrid modulation strategy with phase configurations is adopted. In addition, for spectral reconstruction, a convex optimization framework is employed, in which a differential operator is introduced to decompose the target spectrum into sparse narrowband components and smooth broadband components, thereby achieving high-accuracy reconstruction from the sampled signals. The proposed spectrometer attains a spectral reconstruction resolution of approximately 5 pm over the wavelength range of 1500–1600 nm, while maintaining consistently low reconstruction errors for broadband continuous spectra, sparse spectra, as well as their superimposed hybrid spectral scenarios. These results fully verify the feasibility and superiority of the proposed approach. Overall, this work establishes an integrated framework for high-performance on-chip spectral analysis that combines optimized device architecture, reconfigurable phase modulation, and optimized reconstruction methodologies, providing a promising technological pathway for future applications in portable sensing, wearable health monitoring, and Lab-on-Chip systems.

## 2. Principle and Design

### 2.1. Principle of the Computational Spectrometer

**Figure 1:**
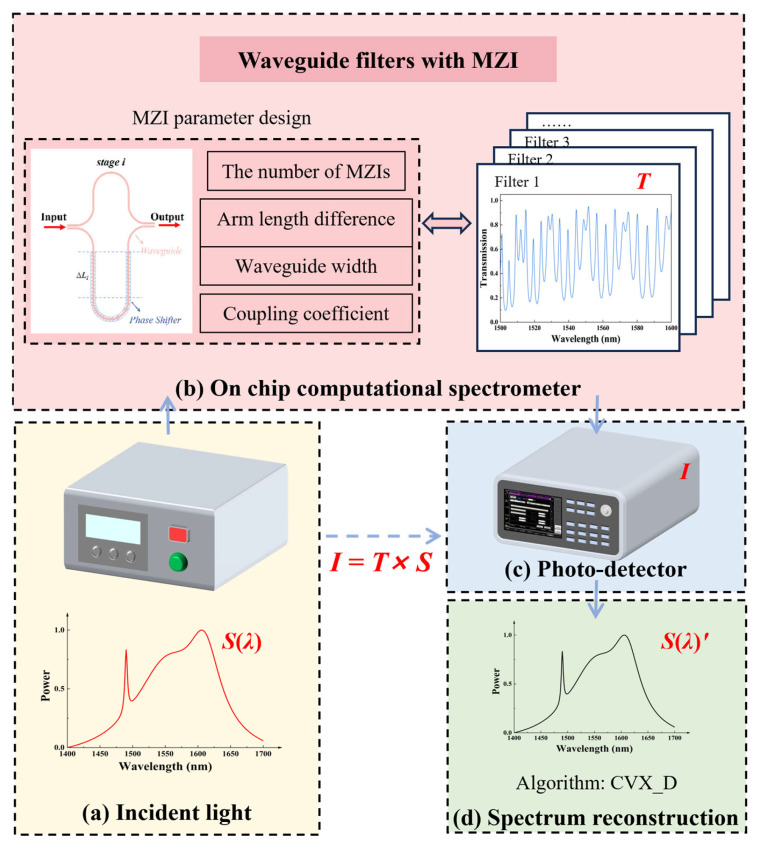
Schematic diagram of the operating principle of the computational spectrometer.

Figure 1 illustrates the operating principle of the computational spectrometer. First, the unknown incident optical signal *S*(*λ*) is modulated and sampled by the sampling unit. Each sampling channel exhibits a distinct optical response function *T_i_*(*λ*), thus encoding the incident spectrum in the spatial or temporal domain, forming the system sampling matrix *T*(*λ*). The sampled optical signals in each channel are converted into a set of intensity signals related to the incident spectrum, and then measured by the photodetector, yielding the output intensity vector *I.* The relationship among these quantities can be expressed as [29]:

(1) I=∫T(λ)S(λ)dλ

After discretization, Equation (1) can be expressed in matrix form as [12]:

(2) $$\left[\begin{array}{c} I_{1}\\ \vdots \\ I_{N} \end{array}\right]=\left[\begin{array}{ccc} T_{1}(\lambda_{1}) & \cdots & T_{1}(\lambda_{M})\\ \vdots & \ddots & \vdots \\ T_{N}(\lambda_{1}) & \cdots & T_{N}(\lambda_{M}) \end{array}\right]\left[\begin{array}{c} S(\lambda_{1})\\ \vdots \\ S(\lambda_{M}) \end{array}\right]$$

where *λ*_1_ and *λ_M_* represent the starting wavelength and ending wavelength of the spectrum, respectively. *M* is the number of spectral pixels in the wavelength domain, and *N* represents the number of sampling channels. Through appropriate design, the condition *N*≪*M* can be achieved in the spectral reconstruction process, which constitutes a key advantage of computational spectrometers. By utilizing a limited number of filters or sampling channels, high-accuracy spectral reconstruction can be achieved, thereby significantly reducing the footprint of the spectrometer chip and meeting the requirements of portable application scenarios.

As can be seen from the above equation, a linear mapping relationship is established between the system output signal *I* and the incident spectrum *S* through the sampling matrix *T*, and the system operates in an underdetermined state. By solving this equation using algorithms such as compressive sensing [30], deep learning [31], and convex optimization [32], the incident spectrum *S* can be reconstructed.

### 2.2. Design of the Computational Spectrometer

As indicated by Equation (2), a linear mapping is established between the system output signal *I* and the incident spectrum *S* through the sampling matrix *T*. Owing to the underdetermined form of the system, where the number of sampling channels *N* is much smaller than the number of spectral sampling points *M*, the accuracy and stability of spectral reconstruction largely depend on the design quality of the sampling matrix *T_N_*_×*M*_. A larger *M* means that a higher spectral resolution can be obtained within a fixed bandwidth or a broader operating bandwidth while maintaining the same resolution. Moreover, an increased *M*/*N* makes it possible to meet a given bandwidth-to-resolution requirement with fewer physical sampling channels. Such a reduction in channel count effectively lowers insertion loss, chip footprint, and overall system complexity. Consequently, for a well-designed computational spectrometer, both the number of wavelength sampling points *M* and the ratio *M*/*N* should be made as large as possible [24].

To simultaneously achieve a large *M* and high *M*/*N*, the spectral responses of the sampling filters must exhibit strong randomness and mutual independence, meaning their auto-correlation (AC) and cross-correlation (XC) should be as small as possible. A low AC indicates that the spectral response of an individual filter is more random, providing denser and more informative sampling points in the wavelength domain, thus improving spectral sampling resolution. Meanwhile, a smaller XC implies reduced correlation and less redundant information among different filters, resulting in a higher rank for the sampling matrix *T_N_*_×*M*_.

(3) $$AC_{i}(\Delta \lambda )=\frac{\left\langle T_{i}(\lambda )T_{i}(\lambda +\Delta \lambda )\right\rangle }{\left\langle T_{i}(\lambda )\right\rangle \left\langle T_{i}(\lambda +\Delta \lambda )\right\rangle }-1$$

(4) $$XC_{ij}(\Delta \lambda )=\frac{\left\langle T_{i}(\lambda )T_{j}(\lambda +\Delta \lambda )\right\rangle }{\left\langle T_{i}(\lambda )\right\rangle \left\langle T_{j}(\lambda +\Delta \lambda )\right\rangle }-1$$

**Figure 2:**
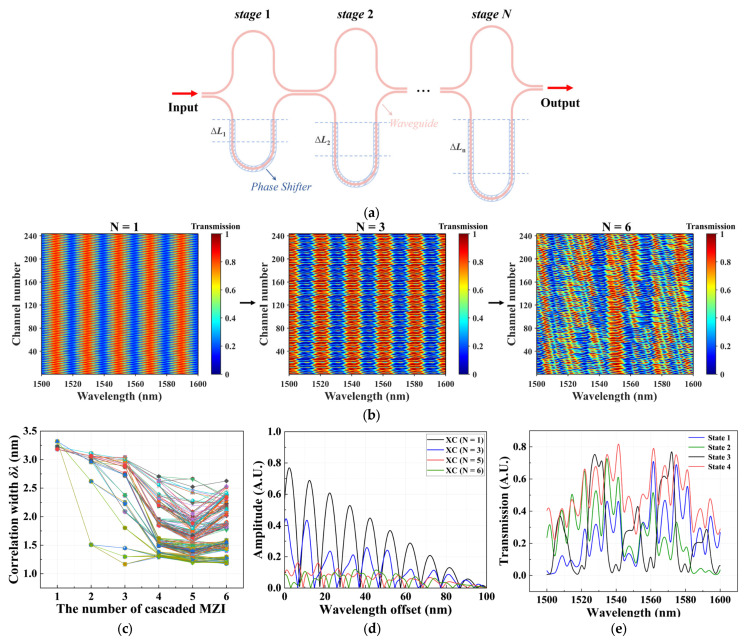
(**a**) Schematic diagram of the proposed on-chip spectrometer based on cascaded MZIs; (**b**) evolution of the simulated transmission spectra with increasing numbers of cascaded MZI stages, forming pseudo-random spectral responses; (**c**) the evolution of the correlation width *δλ* of the spectral auto-correlation function with increasing numbers of cascaded MZI stages; (**d**) the absolute value of the averaged cross-correlation between one specific channel and all the other channels for different numbers of cascaded MZI stages; (**e**) examples of simulated transmission spectra for five-stage cascaded MZIs.

Figure 2a illustrates our proposed spectrometer design, whose core consists of multiple cascaded unbalanced Mach–Zehnder interferometers (MZIs). For an unbalanced MZI, where the optical path lengths of the upper and lower arms satisfy *L*_1_ ≠ *L*_2_, and neglecting device loss, the output optical intensity can be expressed as:

(5) $$I_{out}=\frac{I_{in}}{2}\left[1+\cos(\beta_{1}L_{1}-\beta_{2}L_{2})\right]$$

where *I*_in_ is the input optical intensity, and *β*_1_ and *β*_2_ are the propagation constants of the two interferometer arms, respectively. As indicated by this expression, the output of the interferometer exhibits a periodic dependence on wavelength, with the period defined as the free spectral range (FSR). For a fixed waveguide geometry, the FSR is given by $FSR=\frac{\lambda^{2}}{n_{g}\Delta L}$, where *n_g_* is the waveguide group refractive index, and $\Delta L=|L_{1}-L_{2}|$ is the length difference between the two arms, indicating that the FSR is inversely proportional to the arm-length difference.

Based on the above property, distinct arm-length differences are designed for each MZI in the cascade structure, resulting in mutually different FSRs. When multiple MZIs with different periodic responses are cascaded, the originally regular periodic transmission spectra will be gradually destroyed, finally forming spectral responses with pseudo-random modulation characteristics. Figure 2b shows the evolution of the spectral response for different numbers of cascaded MZIs. As the number of cascaded MZIs increases, the randomness of the transmission spectrum gradually increases. This randomized spectral response significantly improves the system’s capability to encode the incident spectrum and constitutes one of the key factors achieving high-precision spectral reconstruction. Furthermore, the half width at half maximum (HWHM) of the auto-correlation function, referred to as the spectral correlation width *δλ*, can be employed to characterize the channel sampling resolution [33]. Figure 2c presents the relationship between the correlation width *δλ* of the transmission spectrum and the number of cascaded MZI stages. With increasing MZI stages, *δλ* decreases continuously, and the decreasing trend of the first four stages is more significant. When the number of MZIs reaches six, the variation in *δλ* tends to level off. It should be noted, however, that the channel sampling resolution refers only to the overall sampling efficiency of individual channels [13] and cannot fully reflect the joint reconstruction performance of a multi-channel system.

In addition, each MZI integrates a phase shifter, which introduces a controllable additional phase through external voltage adjustment. This enables modulation of the interference condition and consequently alters the transmission response, realizing diversified spectral sampling. It should be noted that the proposed design structure has two output ports, so the system can simultaneously obtain two optical sampling data in each operating state, further improving the utilization efficiency of the sampling channels. By applying different voltage configurations to the phase shifters at each stage, a large number of mutually uncorrelated sampling channels can be generated, providing sufficient data support for subsequent spectral reconstruction. The number of channels increases exponentially with the system parameters. Specifically, for a cascaded structure with *N* MZI stages and *n* phase states per stage, the total number of sampling channels *N_channel_* follows:

(6) $$N_{channel}=2\times n^{N}$$

Figure 2d shows the cross-correlation (XC) results among the sampling channels generated by cascaded MZIs with different stage numbers. As the number of MZI stages increases, the cross-correlation values are significantly reduced. When the MZI consists of five or six stages, the cross-correlation remains below 0.2, indicating that the proposed structure can effectively generate highly independent sampling channels. This property provides strong support for the stability and accuracy of the subsequent spectral reconstruction algorithm. Considering that further increasing the stage number would introduce additional structural complexities and tuning difficulties, the MZI stage number was ultimately set to five. Figure 2e presents the simulated transmission spectra of the five-stage cascaded MZI, where each MZI stage is configured with two discrete phase states (0 and π/4), under several representative phase-configuration conditions.

### 2.3. Optimization of Reconstruction Algorithms

The incident spectrum *S* can be reconstructed by minimizing the L_2_-norm in Equation (2) with respect to *S*′ using a convex optimization algorithm [32,34,35]:

(7) $$Minimize\;\;{\left\|I-T\cdot S^{\prime }\right\|}_{2}^{2}$$

where S’ denotes the reconstructed incident spectrum obtained by solving the above minimization problem. In practice, the measured spectra may exhibit diverse characteristics, including sparse narrowband spectral lines with local sharp features, as well as broadband spectral components with smooth and continuous variations. Relying solely on Equation (7), it is difficult to achieve high-accuracy reconstruction results. To address this issue, this work models spectral signals according to their inherent structural characteristics and introduces appropriate regularization strategies for different spectral components, thereby enhancing the stability and reliability of the spectral reconstruction.

Accordingly, we decompose the spectrum *S* to be measured into two components from the structural characteristics of spectral signals: a sparse narrowband signal *S*_1_ and a smooth broadband signal *S*_2_, given by:

(8) $$S=S_{1}+S_{2}$$

The component *S*_1_ characterizes locally sharp spectral lines or narrowband signal components (such as absorption peaks and emission lines) in the spectrum, while component *S*_2_ corresponds to the background or broadband continuous spectral components that vary slowly with wavelength. Together, these two components form complementary structural features of the spectral signal. Based on this decomposition, to properly accommodate the essential differences between the two components, we designed a differentiated regularization constraint strategy: for the sparse component *S*_1_, the L_1_-norm regularization term is introduced to enhance the sparsity of the solution; while for the smooth component *S*_2_, a smoothness constraint is constructed using the differential operator *D,* which is expressed as:

(9) $$D\cdot S_{2}=S_{2}(\lambda_{i+1})-S_{2}(\lambda_{i})$$

The primary function of the differential operator is to quantitatively describe the variation amplitude of the spectral signal between adjacent sampling points, and *D* is an N × N matrix, where N donates the number of sampling channels. By minimizing its L_2_-norm, the naturally smooth variation in the background spectrum is preserved to the greatest extent. Compared with the traditional Tikhonov regularization or directly applying the L_2_-norm constraint on the entire spectrum [36], the proposed joint regularization scheme, which combines sparse regularization (L_1_-norm) with smoothness regularization (L_2_-norm of the difference operator), realizes a precise matching and coordinated constraint of sparse and smooth spectral features. Specifically, the L_1_ regularization effectively exploits the intrinsic sparsity of narrowband spectral lines, whereas the smoothness constraint induced by the difference operator preserves the continuous structure of the broadband background spectrum. This complementary mechanism avoids the limitations of single regularization strategies, such as insufficient sparsity promotion or the excessive smoothing of details that may lead to information loss, thereby significantly improving the accuracy and robustness of the overall spectral reconstruction results.

By integrating the aforementioned strategies, the improved spectral reconstruction model can be formulated as the following minimization problem with respect to *S*_1_′ and *S*_2_′:

(10) $$Minimize\;\;{\left\|I-T\cdot \left(S_{1}^{\prime }+S_{2}^{\prime }\right)\right\|}_{2}^{2}+\gamma_{1}{\left\|S_{1}^{\prime }\right\|}_{1}+\gamma_{2}{\left\|D\cdot S_{2}^{\prime }\right\|}_{2}$$

where $S^{\prime }=S_{1}^{\prime }+S_{2}^{\prime }$, *γ*_1_ and *γ*_2_ are weighting coefficients that adjust the relative strengths of the sparse regularization and the differential smoothness regularization terms, respectively. The L_1_-norm penalty ${\left\|S_{1}^{\prime }\right\|}_{1}$ is imposed to promote sparsity of the narrowband spectral component, whereas the L_2_-norm term ${\left\|D\cdot S_{2}^{\prime }\right\|}_{2}$ enforces smoothness of the broadband component through the difference operator *D*. In practice, the values of *γ*_1_ and *γ*_2_ were determined empirically via cross-validation [37]. Specifically, in this work, a logarithmic grid search was performed over candidate values ranging from 10^−4^ to 10^0^, and the optimal pair (*γ*_1_, *γ*_2_) was selected by minimizing the spectral reconstruction error. This procedure ensures a balanced trade-off between sparsity enforcement and the smoothness constraint, thereby improving reconstruction fidelity while avoiding overfitting.

With this decomposition modeling and joint regularization approach, the proposed algorithm can simultaneously achieve accurate reconstructions of sparse spectral lines and smooth recoveries of broadband backgrounds, significantly improving reconstruction accuracy and robustness under complex spectral profiles.

## 3. Experiment

### 3.1. Device Design and Characterization

**Figure 3:**
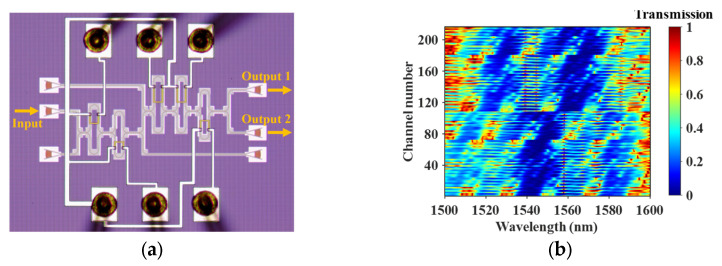

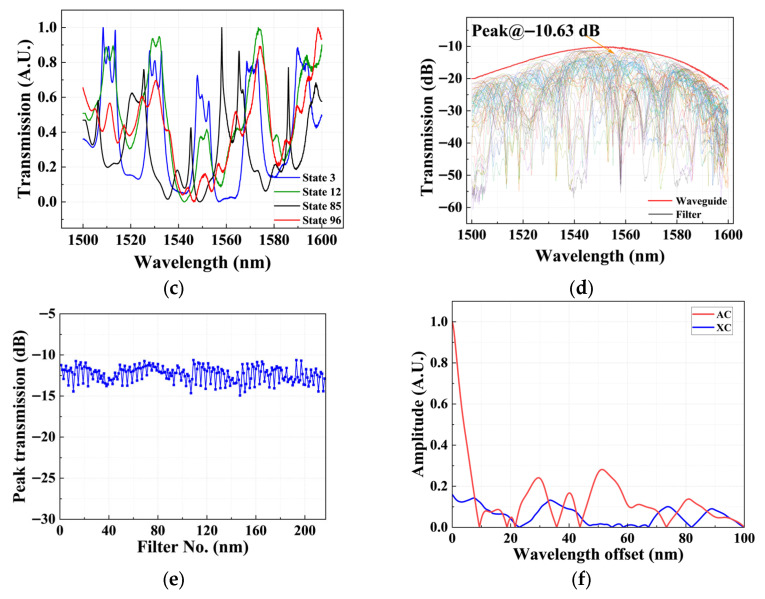
(**a**) Image of the on-chip spectrometer based on cascaded MZIs; (**b**) heat map of the measured sampling matrix *T*; (**c**) measured transmission spectra in linear scale of four different operating states; (**d**) measured transmission spectra in log scale; (**e**) peak transmissions of the 216 channels; (**f**) auto-correlation and cross-correlation of transmission spectra under 108 sampling states (216 sampling channels) of the on-chip spectrometer.

The device is fabricated on a silicon-on-insulator (SOI) platform, employing strip waveguides with a height of 220 nm and a width of 450 nm. Owing to its high refractive-index contrast and full compatibility with mature CMOS fabrication processes, the SOI platform enables compact integration of multi-stage MZIs within a limited chip footprint while maintaining low bending loss and stable phase modulation characteristics. The chip fabrication was carried out by Chongqing United Microelectronics Co., Ltd. (CUMEC, Chongqing, China). Figure 3a shows the designed and fabricated five-stage cascaded MZI device in this work, with a chip footprint of 550 × 500 µm^2^. To satisfy the requirements of randomness and mutual independence in the sampling system, the free spectral ranges (FSRs) of the cascaded MZIs are deliberately designed to be non-overlapping. Specifically, one arm length of each MZI is fixed at 100 μm, while the other arm has different lengths in different stages, introducing distinct arm-length differences ΔL_i_. Under the constraints of fabrication feasibility and propagation loss, the values of ΔL are selected to ensure that the transmission spectrum of the cascaded MZI exhibits sufficient spectral diversity over the entire operating bandwidth, thereby improving the rank of the sampling matrix and its pseudo-random characteristics.

In terms of phase modulation, based on simulation results and considering the trade-off between system complexity and the number of sampling channels, a hybrid modulation strategy is adopted in this work: the phase shifters of the first three MZI stages are configured with three discrete operating states (0, π/4, and π/2), while those in the last two stages are set to two states (0 and π/4). As a result, a total of 3^3^ × 2^2^ = 108 distinct operating states are generated. Taking advantage of the two complementary output ports of the proposed structure, 216 different sampling filter transmission responses are ultimately obtained and employed to construct the sampling matrix *T*. Under this configuration, the power consumptions of the five-stage MZI phase shifters are 26.59 mW, 21.02 mW, 28.09 mW, 14.92 mW, and 13.32 mW, respectively, resulting in a total power consumption of 103.94 mW. This design provides a sufficiently large configurable phase-combination space under limited driving power consumption.

During the experimental characterization, a tunable laser source (N7776C, Keysight Technologies, Santa Rosa, CA, USA) was used to provide the input optical signal, and the output power was monitored using an optical power meter (N7745C, Keysight Technologies, Santa Rosa, CA, USA), enabling the measurement of the transmission spectra under different modulation states. Figure 3b presents the heat map of the sampling matrix *T* composed of the experimentally measured transmission spectra of the 216 sampling channels. Figure 3c and Figure 3d plot the experimental results of the filters’ spectral responses in linear and log scale, respectively. The insertion losses of the filters are analyzed. It is observed that the peak transmissions of the filters are around −10.63 dB. The total loss from laser to detector includes setup loss (fiber connectors, polarization controllers, etc.), fiber/chip coupling losses and the spectrometer’s insertion loss (from input to output). The previous two losses amount to about 10.13 dB according to the transmission curve of a straight waveguide with two grating couplers, indicating that the spectrometer’s insertion loss is approximately 0.5 dB. Consequently, the loss per individual MZI is around 0.1 dB. Figure 3f shows the auto-correlation and cross-correlation of all channels. The cross-correlation values are all below 0.2, indicating good decorrelation among sampling channels, laying a solid foundation for achieving high-precision spectral reconstruction.

### 3.2. Spectral Reconstruction Results

Combining the test results of the designed on-chip spectrometer with the CVX-based reconstruction algorithm, different types of spectra are reconstructed. The reconstruction performance is evaluated using root mean squared error (RMSE) and relative error *ε*, defined as follows:

(11) $$RMSE=\sqrt{\frac{{\sum_{i=1}^{m}\left(S-S^{\prime }\right)}^{2}}{m}}$$

(12) $$\varepsilon =\frac{\|S-S^{\prime }{\|}_{2}}{\|S{\|}_{2}}$$

**Figure 4:**
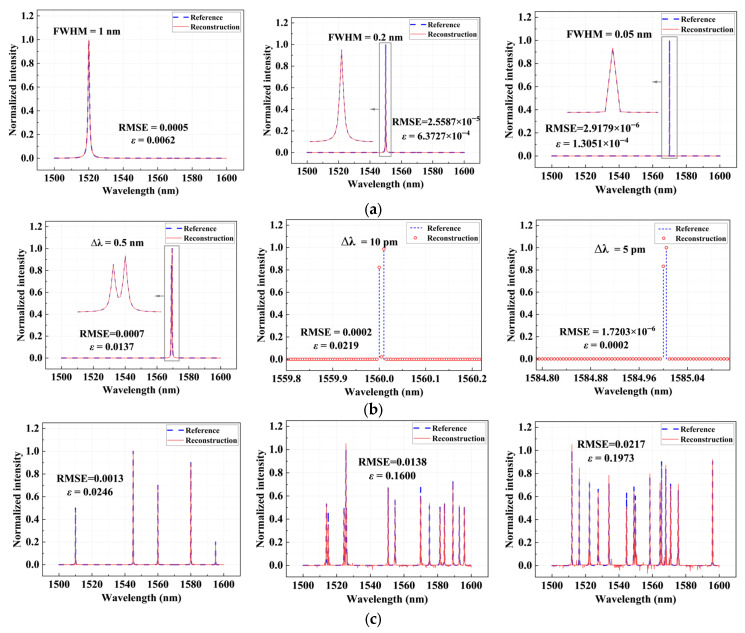
Sparse spectral reconstruction results. (**a**) Single-peak spectral reconstruction with different center wavelengths and FWHM; (**b**) Dual-peak spectral reconstruction with different wavelength intervals; (**c**) multi-peak spectral reconstruction. The blue dashed curves represent the original spectra, while the red solid curves denote the reconstructed spectra. The arrows point to the regions of the spectra that are enlarged in the boxed insets.

Figure 4 shows the sparse spectrum reconstruction results under different center wavelengths and full width at half maximum (FWHM) conditions over a broad wavelength range from 1500 to 1600 nm. First, in the single-peak spectrum reconstruction experiments, narrowband spectral lines with FWHMs of 1 nm, 0.2 nm, and 0.05 nm are constructed, respectively. The reconstruction results demonstrate that the proposed scheme maintains an exceptionally high reconstruction accuracy. The root mean squared error (RMSE) of the reconstructed spectra remains below 0.0005, and the relative error ε is less than 0.0062, indicating negligible reconstruction errors. These results demonstrate that the proposed spectrometer can not only accurately locate the unknown spectral peak positions but also faithfully capture their peak amplitudes.

To further evaluate the spectral resolving capability, dual-peak spectrum reconstruction experiments with different wavelength intervals are conducted, as shown in Figure 4b. The intervals between the two spectral lines are set to 0.5 nm, 0.01 nm, and an extremely small value of 0.005 nm, respectively. Even at an interval of 0.005 nm, the *ε* of the reconstruction is only 0.0002. The two spectral lines can still be clearly distinguished, conclusively demonstrating that the designed chip can achieve a spectral reconstruction resolution of 5 pm over a 100 nm operating bandwidth. Furthermore, we test the reconstruction performance of multi-peak spectra, including combinations of multiple spectral lines with different intensities and positions, as shown in Figure 4c. Under these more complex spectral conditions, the overall RMSE ranged from 0.0013 to 0.0217. The reconstruction spectra still clearly resolve the positions and relative amplitudes of all spectral peaks, indicating that the spectrometer maintains stable accuracies for complex spectral components and exhibits good generality.

**Figure 5:**
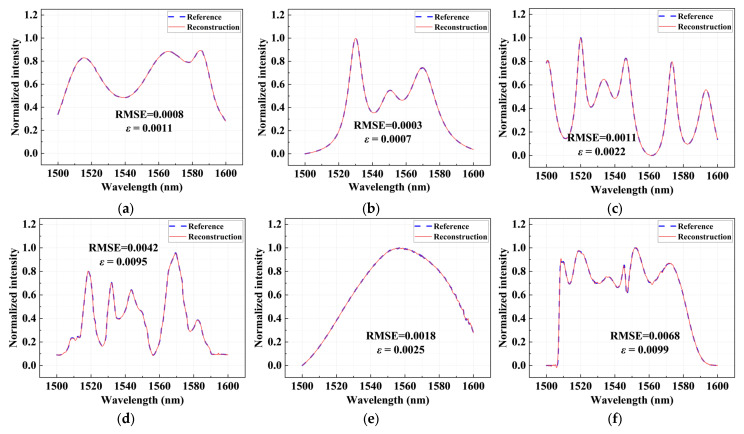
Continuous broadband spectral reconstruction results. (**a**–**d**) Broadband spectra synthesized by superposition of multiple Lorentzian functions with different center wavelengths and FWHMs; (**e**,**f**) ASE source spectrum. The blue dashed curves represent the original spectra, while the red solid curves denote the reconstructed spectra.

After verifying the reconstruction performance of sparse spectra, the reconstruction capability of the proposed spectrometer under broadband conditions is further evaluated. Compared with sharp spectral lines, broadband signals exhibit flatter and more continuous energy distributions, thereby imposing higher demands on the smooth response of the system. To emulate more realistic and complex spectral scenarios, broadband spectra synthesized by the superposition of multiple Lorentzian functions with different center wavelengths and FWHMs are constructed. As shown in Figure 5, the reconstructed spectra show excellent agreement with the original spectra in terms of overall envelope, local details, and intensity distribution, with RMSE ranging from 0.0003 to 0.0042 and relative errors between 0.0007 and 0.0095, indicating that the designed spectral chip maintains high reconstruction accuracies even for complex continuous broadband signals.

Furthermore, the proposed spectrometer is further verified by reconstructing the measured spectra of two amplified spontaneous emission (ASE) sources, as shown in Figure 5e,f. For these two experimental spectra, the reconstruction RMSEs are 0.0018 and 0.0068, respectively, and the relative errors *ε* are 0.0025 and 0.0099. This further demonstrates that the spectrometer not only performs excellently under simulation conditions but can also accurately reconstruct the broadband spectra of practical light sources, exhibiting strong generalization abilities and application potential.

After verifying the independent reconstruction capabilities of sparse spectra and broadband spectra, we further examined the performance of the proposed spectrometer under more complex scenarios, namely hybrid spectra composed of a smooth, continuous broadband background superimposed with narrowband characteristic peaks. Such spectra are widely found in practical applications, such as characteristic emission lines embedded in complex background signals, or absorption spectra acquired from broadband signals, which simultaneously contain low-frequency and high-frequency components. This places higher demands on the resolution and robustness of the reconstruction algorithm.

**Figure 6:**
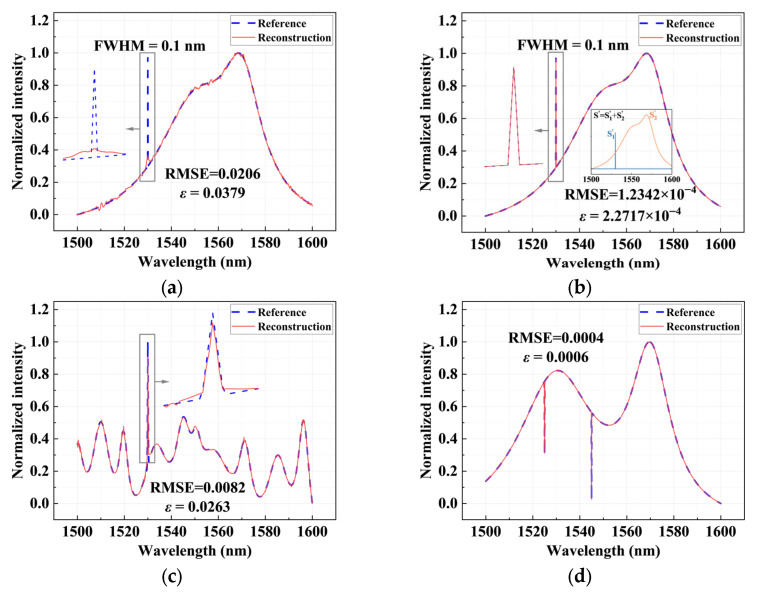
Hybrid spectral reconstruction results. (**a**) Reconstruction obtained by directly applying regularization to the entire spectrum; (**b**–**d**) reconstruction results using the proposed decomposition-based regularization approach. Two types of hybrid spectra are reconstructed, consisting of a broadband background superimposed with locally sharp features: convex peaks in (**b**,**c**) and concave dips in (**d**). For the inset in (**b**), $S_{1}^{\prime }$ and $S_{2}^{\prime }$ denote the reconstructed spectra of the sparse narrowband and smooth broadband components, respectively. The blue dashed curves represent the original spectra, while the red solid curves denote the reconstructed spectra. The arrows point to the regions of the spectra that are enlarged in the boxed insets.

Figure 6a shows the hybrid spectrum of a broadband background superimposed with a narrowband peak of 0.1 nm FWHM. Figure 6a also illustrates the reconstruction result obtained using a conventional approach that directly applies regularization to the entire spectrum. In this case, the narrow peak is significantly suppressed or even completely flattened due to the smoothness constraint, failing to recover the sharp spectral feature. In contrast, when the proposed decomposition-based reconstruction strategy is employed, as shown in Figure 6b, the reconstruction error is markedly reduced, with an RMSE of only 1.2342 × 10^−4^, and the reconstructed spectrum exhibits excellent agreement with the original input spectrum. By decomposing the spectrum into a sparse narrowband component *S*_1_ and a smooth broadband component *S*_2_, the inset in Figure 6b further presents the two reconstructed components separately. The narrow peak is completely preserved, and the broad background maintains its original variation trend, clearly demonstrating the significant advantage of this strategy in hybrid spectral reconstruction.

Under more challenging conditions with increasingly complex backgrounds—characterized by pronounced fluctuations, non-monotonic behavior, and richer spectral variations—the proposed algorithm remains robust. As shown in Figure 6c, both the broadband and narrowband components are stably separated and accurately reconstructed, without observable artifacts such as peak drift, broadening, or intensity distortion. In addition, the reconstruction performance for concave-feature hybrid spectra is also evaluated, as shown in Figure 6d. The proposed method is still able to precisely reconstruct both the positions and depths of the two valleys. The reconstructed curve shows an almost perfect overlap with the original spectrum, achieving an RMSE of 0.0004 and a relative error of 0.0006.

Overall, the above results show that the proposed spectroscopic chip can not only effectively handle sparse peaks and broadband continuous spectra but also maintain excellent reconstruction performance in more complex hybrid spectral scenarios. This favorable adaptability to diverse spectral types reflects the generality and robustness of the system across different spectral structures. In addition, the reconstruction latency is evaluated under the configuration of 216 sampling channels. The proposed algorithm completes a full spectral reconstruction within approximately 2.4 s per spectrum on a personal computer equipped with an Intel Core i7-14700HX CPU (Intel, Santa Clara, CA, USA). This latency can be further reduced through parallel computation or hardware acceleration, enabling practical deployment in rapid spectral sensing applications.

## 4. Discussion

**Figure 7:**
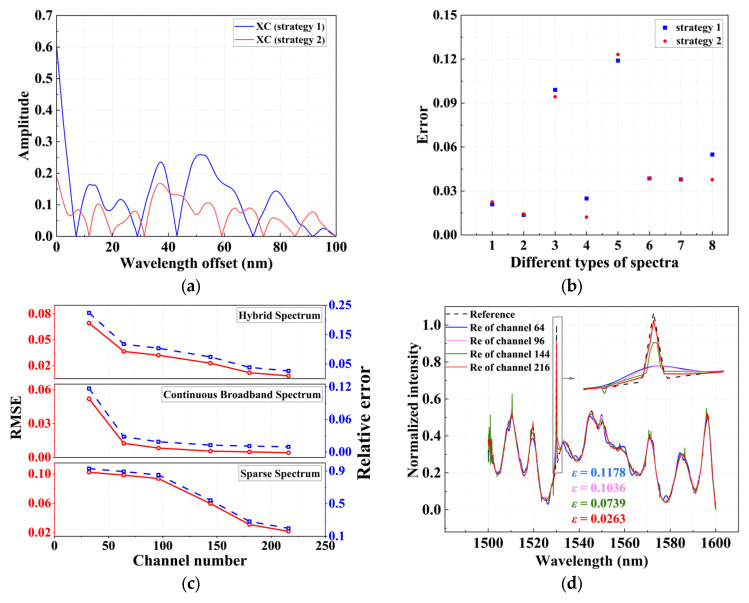
(**a**) Cross-correlation comparison under two different phase-configuration strategies; (**b**) reconstruction relative errors for different spectra under the two phase-configuration strategies; (**c**) variations in RMSE and relative error with respect to the number of sampling channels for different types of spectra; (**d**) Spectral reconstruction results obtained with different numbers of sampling channels. The arrows point to the regions of the spectra that are enlarged in the boxed insets.

To further investigate the impact of phase shifter configurations on the independence of sampling channels, a comparative experiment is conducted using two different phase-setting strategies. In the first strategy, all five phase shifters are configured with the same two operating states, corresponding to phase shifts of Δ*φ =* 0 or π/4, resulting in 32 operating states. In the second strategy, distinct phase settings are assigned to different phase shifters, with phase shifts given by Δ*φ*_1_ = 0 or π, Δ*φ*_2_ = π/6 or 2π/3, Δ*φ*_3_ = π/3 or 5π/6, Δ*φ*_4_ = π/5 or 3π/5, Δ*φ*_5_ = 2π/5 or 4π/5, also resulting in 32 operating states. The output transmission spectra for both strategies are measured and analyzed. Figure 7a shows the cross-correlation comparison results among the transmission spectra of the 32 sampling channels under the two phase-configuration strategies. It can be seen that when all phase shifters employ identical phase variations, the cross-correlation between different channels remains relatively high. In contrast, after adopting a hierarchical and differentiated phase setting, the cross-correlation values are consistently below 0.2, indicating stronger decorrelation. Figure 7b presents a comparison of the reconstruction errors obtained under two phase-configuration strategies for various spectral reconstruction tasks. It can be observed that, in most cases, the hierarchical differentiated phase setting achieves lower reconstruction errors, indicating generally superior reconstruction performance.

Besides the phase-configuration method, the number of sampling channels is also a critical factor affecting spectral reconstruction accuracy and plays a vital role in determining overall system performance. Figure 7c illustrates the variations in the root mean squared error (RMSE) and relative error *ε* for the reconstruction of three different types of spectra under varying numbers of sampling channels. As the channel count increases from 32 to 216, the relative reduction in reconstruction error for different types of spectra is 88.3%, 19.1%, and 78.8%, respectively, demonstrating the dominant influence of channel number on reconstruction accuracy. Figure 7d compares the spectral reconstruction performance of the hybrid spectrometer under different numbers of sampling channels. As the channel number increases from 64 to 216, the discrepancy between the reconstructed spectrum and the original spectrum becomes progressively smaller, and the reconstruction relative error *ε* continuously decreases. When the number of channels reaches 216, the reconstructed spectrum almost perfectly overlaps with the original one, indicating a significant improvement in reconstruction accuracy. Specifically, in the 64-channel case, each phase shifter is configured with only two discrete phase states. By progressively introducing additional phase states for each phase shifter, the number of sampling channels increases accordingly. When the channel count is extended to 216, the first three phase shifters are assigned three phase states, while the remaining two retain two states, resulting in a substantially richer set of sampling responses. This phase-setting strategy enhances reconstruction performance without excessively increasing hardware complexity, thereby achieving an effective trade-off between the number of sampling channels and spectral reconstruction accuracy.

## 5. Conclusions

In this work, a reconfigurable silicon photonic on-chip spectrometer based on a multi-stage cascaded Mach–Zehnder interferometer (MZI) is proposed and experimentally demonstrated. A five-stage cascaded MZI architecture with distinct arm-length differences is designed and fabricated on an SOI platform. Combined with phase modulation, a spectral response containing 216 sampling channels is constructed. Experimental results show that the proposed structure can generate transmission spectra with pseudo-random characteristics over the wavelength range from 1500 to 1600 nm, with cross-correlation values among all sampling channels below 0.2. For spectral reconstruction, a difference-operator decomposition strategy is employed to separate the target spectrum into sparse narrowband and smooth broadband components, which are then reconstructed using a convex-based algorithm. The results demonstrate that the proposed spectrometer achieves excellent reconstruction performances for sparse spectra, continuous broadband spectra, and complex hybrid spectra composed of both components. The reconstruction RMSE is below 0.01 and the relative error *ε* is less than 0.03, with reconstruction errors reaching the 10^−6^ level for certain spectral cases. Under the current configuration with a five-stage MZI and 216 sampling channels, a spectral reconstruction resolution of 5 pm is achieved over a 100 nm operating bandwidth. According to specific application requirements, the number of sampling channels, reconstruction accuracies, and spectral resolutions can be flexibly tailored by adjusting the number of cascaded MZI stages or by reconfiguring the phase settings among different stages, thereby enabling higher-performance system configurations.

In summary, the proposed reconfigurable computational spectrometer exhibits excellent overall performances in terms of the device architecture, phase modulation strategy, and reconstruction algorithm, providing a feasible and scalable technical pathway for achieving low-power, small-footprint, and high-precision on-chip spectral analysis.

## Figures and Tables

**Table 1:** Performance comparison of computational reconstruction spectrometers.

Type	Ref.	Footprint/µm^2^	Bandwidth/nm	Resolution/nm	Number of Channels
Multimode waveguide	[17]	500 × 500	2	0.01	40
	[18]	1600 × 2100	2	0.016	121
	[19]	--	6.4	0.16	64
	[20]	1.5 × 10^6^ (With on-chip photodetectors)	100	0.02	384
Disordered media	[21]	50 × 100	25	0.75	25
	[22]	100 × 200	50@1550 nm 15@800 nm	4@1550 nm 0.03@800 nm	16
	[23]	100 × 200	40@1550 nm 15@775 nm	3@1550 nm 0.3@775 nm	13
Waveguide filter type	[11]	2000 × 7600	115	0.03	256
	[12]	40 × 100 (Based on simulation)	200	0.1~2	192~2187
	[13]	520 × 220	12	0.02	64
	[14]	35 × 260	180	0.45	32
	[24]	3 × 10^6^	100	0.1	64
	[25]	60 × 60	100	0.04	2501
	[26]	2900 × 3700	200	0.01	729
	[27]	600 × 800	40	0.06	64
	[28]	5 × 10^5^	100	0.01~0.3	132
	Our work	550 × 500	100	0.005	216

## Data Availability

Data underlying the results presented in this paper are available from the authors upon reasonable request.

## Abbreviations

The following abbreviations are used in this manuscript:

MZI	Mach–Zehnder Interferometer
AC	Auto-correlation
XC	Cross-correlation
FSR	Free Spectral Range
HWHM	Half Width at Half Maximum
SOI	Silicon-on-insulator
CMOS	Complementary Metal Oxide Semiconductor
CVX	Convex Optimization
RMSE	Root Mean Squared Error
FWHM	Full Width at Half Maximum
